# Dose–Response Effects of Patient Engagement on Health Outcomes in an mHealth Intervention: Secondary Analysis of a Randomized Controlled Trial

**DOI:** 10.2196/25586

**Published:** 2022-01-04

**Authors:** Yiran Li, Yan Guo, Y Alicia Hong, Yu Zeng, Aliza Monroe-Wise, Chengbo Zeng, Mengting Zhu, Hanxi Zhang, Jiaying Qiao, Zhimeng Xu, Weiping Cai, Linghua Li, Cong Liu

**Affiliations:** 1 Department of Medical Statistics School of Public Health Guangzhou China; 2 Sun Yat-sen Center for Global Health Guangzhou China; 3 Department of Population and Quantitative Health Sciences University of Massachusetts Chan Medical School Worcester, MA United States; 4 Department of Health Administration and Policy College of Health and Human Services George Mason University Fairfax, VA United States; 5 Department of Global Health University of Washington Seattle, WA United States; 6 South Carolina SmartState Center for Healthcare Quality Arnold School of Public Health University of South Carolina Columbia, SC United States; 7 Department of Health Promotion, Education, and Behavior Arnold School of Public Health University of South Carolina Columbia, SC United States; 8 The Jockey Club School of Public Health and Primary Care Faculty of Medicine The Chinese University of Hong Kong Hong Kong Hong Kong; 9 National Center of AIDS/STD Control and Prevention Chinese Center for Disease Control and Prevention Beijing China; 10 Department of Infectious Diseases Guangzhou Eighth People’s Hospital, Guangzhou Medical University Guangzhou China

**Keywords:** mHealth, patient engagement, dose–response relationship, long-term effect, generalized linear mixed effects model

## Abstract

**Background:**

The dose–response relationship between patient engagement and long-term intervention effects in mobile health (mHealth) interventions are understudied. Studies exploring long-term and potentially changing relationships between patient engagement and health outcomes in mHealth interventions are needed.

**Objective:**

This study aims to examine dose–response relationships between patient engagement and 3 psychosocial outcomes in an mHealth intervention, Run4Love, using repeated measurements of outcomes at baseline and 3, 6, and 9 months.

**Methods:**

This study is a secondary analysis using longitudinal data from the Run4Love trial, a randomized controlled trial with 300 people living with HIV and elevated depressive symptoms to examine the effects of a 3-month mHealth intervention on reducing depressive symptoms and improving quality of life (QOL). We examined the relationships between patient engagement and depressive symptoms, QOL, and perceived stress in the intervention group (N=150) using 4–time-point outcome measurements. Patient engagement was assessed using the completion rate of course assignments and frequency of items completed. Cluster analysis was used to categorize patients into high- and low-engagement groups. Generalized linear mixed effects models were conducted to investigate the dose–response relationships between patient engagement and outcomes.

**Results:**

The cluster analysis identified 2 clusters that were distinctively different from each other. The first cluster comprised 72 participants with good compliance to the intervention, completing an average of 74% (53/72) of intervention items (IQR 0.22). The second cluster comprised 78 participants with low compliance to the intervention, completing an average of 15% (11/72) of intervention items (IQR 0.23). Results of the generalized linear mixed effects models showed that, compared with the low-engagement group, the high-engagement group had a significant reduction in more depressive symptoms (*β*=−1.93; *P*=.008) and perceived stress (*β*=−1.72; *P*<.001) and an improved QOL (*β*=2.41; *P*=.01) over 9 months. From baseline to 3, 6, and 9 months, the differences in depressive symptoms between the 2 engagement groups were 0.8, 1.6, 2.3, and 3.7 points, respectively, indicating widening between-group differences over time. Similarly, between-group differences in QOL and perceived stress increased over time (group differences in QOL: 0.9, 1.9, 4.7, and 5.1 points, respectively; group differences in the Perceived Stress Scale: 0.9, 1.4, 2.3, and 3.0 points, respectively).

**Conclusions:**

This study revealed a positive long-term dose–response relationship between patient engagement and 3 psychosocial outcomes among people living with HIV and elevated depressive symptoms in an mHealth intervention over 9 months using 4 time-point repeat measurement data. The high- and low-engagement groups showed significant and widening differences in depressive symptoms, QOL, and perceived stress at the 3-, 6-, and 9-month follow-ups. Future mHealth interventions should improve patient engagement to achieve long-term and sustained intervention effects.

**Trial Registration:**

Chinese Clinical Trial Registry ChiCTR-IPR-17012606; https://www.chictr.org.cn/showproj.aspx?proj=21019

## Introduction

### Background

Mobile health (mHealth) interventions have gained increasing momentum in delivering easily accessible, patient-centered, individually tailored, and potentially cost-effective programs for a range of psychosocial disorders [1-3]. Previous studies have demonstrated the effectiveness of mHealth interventions in improving psychological outcomes [4,5]. For example, internet-based cognitive behavioral therapy (CBT) has been proven to be effective in treating depressive symptoms [4]. Another study found that an mHealth intervention with self-care strategies reduced depressive symptoms in people living with HIV [5]. However, few studies have explored the impact of patient engagement with mHealth interventions on long-term patient outcomes. Patient engagement is defined as the degree to which a patient adheres to an intervention [6]. Examining the impact of patient engagement on intervention effects beyond the termination of the intervention could help better understand the dose–response relationship in interventions.

Limited mHealth studies have examined the dose–response relationship in program evaluation. Of the few such studies, the most used is the pre–post design with short-term follow-up, typically within 3 months of the intervention [7-10]. To the best of our knowledge, there is only 1 mHealth study that aimed to explore the long-term dose–response relationship between patient engagement and mental health outcomes over 9 months [11]. This 3-month computerized CBT intervention found that different measures of patient engagement, such as number of log-ins, total time spent on the program, and number of visits to a mood diary (1 component of the intervention), were significantly associated with reduced depressive symptoms immediately after the intervention. In the long term, the completion rate of homework assignments was a significant predictor of reduced depressive symptoms at 9 months. However, this study only measured outcomes at the 3- and 9-month follow-ups, and the logistic regression used in the study could not reveal the likely changing dose–response relationship over time. Longitudinal studies with repeated measurements (≥3) may allow us to examine the changing relationship between patient engagement and intervention effects over time. A better understanding of the potential time-varying relationship between patient engagement and mHealth intervention effects is warranted in the long term.

Long-term dose–response relationships have been examined more thoroughly in face-to-face interventions than in mHealth interventions. In face-to-face CBT interventions, homework assignments are considered indispensable to the effect of psychotherapy. Homework is defined as structured, specific, and therapeutic activities that are routinely completed by the participants between sessions. Homework tasks might include self-monitoring of mood, thoughts and behaviors, behavioral activation, or specific cognitive and behavioral skills, such as breathing exercises [12,13]. Studies have found that homework compliance has a positive impact on psychosocial outcomes, such as reduction of anxiety or depressive symptoms at 6 or 12 months after treatment in face-to-face CBT interventions [12,13]. Compared with face-to-face interventions, mHealth interventions allow for easy and repeated access to intervention materials for participants long after the formal intervention period, which may result in long-term and sustained intervention effects. Understanding the potential impacts of long-term patient engagement with interventions and its associated outcomes is crucial for progress tracking, intervention refinement, and future scale-up for mHealth interventions.

Furthermore, patient engagement in mHealth interventions may be different from face-to-face interventions as the former captures more multi-faceted aspects of patient engagement, such as log-ins, completion rate, frequency of items completed, and time spent on the program [7,14]. Our previous study examined the associations between patient engagement and intervention outcomes at 3 months in the Run4Love program. We found that a higher completion rate and a greater frequency of completed items were associated with fewer depressive symptoms at 3 months [15]. These automated and multi-dimensional patient engagement data may provide important insights into intervention progress tracking and interpretation of intervention mechanisms. Given that mHealth tools allow for the capture of more multi-faceted factors of patient engagement, more studies to evaluate the impacts of patient engagement on health outcomes in mHealth interventions are needed [14,16,17].

### Objectives

This study aims to examine the potential time-varying dose–response relationships between patient engagement and intervention effects in a randomized controlled trial of an mHealth intervention, the Run4Love program, and to fill gaps in the literature. The Run4Love trial aimed to reduce stress and depressive symptoms and to improve quality of life (QOL) among people who lived with HIV and were concurrently experiencing elevated depressive symptoms. We estimated the impact of patient engagement on depressive symptoms (the primary outcome of the intervention), QOL, and perceived stress at the 3-, 6-, and 9-month follow-ups. We hypothesize that better patient engagement in an mHealth intervention could lead to better and sustained health outcomes in the long term.

## Methods

### Overview

This study is a secondary analysis using data from the Run4Love trial, a parallel randomized controlled trial, to examine the effects of a WeChat (Tencent Holdings Limited)-based intervention on reducing depressive symptoms in people living with HIV and elevated depressive symptoms. The study design and primary results of the Run4Love trial have been published elsewhere [18,19]. The trial was registered in the Chinese Clinical Trial Registry (ChiCTR-IPR-17012606). The study protocol was approved by the Institutional Review Board of Sun Yat-sen University and has been published [19].

### Participants and Procedure

A total of 300 people living with HIV and elevated depressive symptoms were recruited from the outpatient department of a large hospital designated for HIV treatment in Guangzhou, the third largest city in China, in 2017. The participants were recruited if they (1) were aged ≥18 years, (2) were HIV-seropositive, (3) had elevated depressive symptoms (measured using a Center for Epidemiologic Studies-Depression [CES-D] score of ≥16), (4) were active users of WeChat, and (5) were willing to provide hair samples (to measure cortisol as a biomarker of chronic stress). Participants were excluded if they were (1) currently on psychiatric or psychological treatment, (2) unable to finish the questionnaire, and (3) unable to engage in the intervention (read or listen to the materials on WeChat or engage in physical exercise because of medical or other reasons). The participants who met the eligibility criteria and were willing to participate completed a baseline survey and were randomized into the intervention or waitlist control group. A total of 150 participants in the intervention group received a 3-month Run4Love intervention and a 3-month booster session; the participants in the control group received a brochure on HIV-related nutrition in addition to usual care for HIV treatment. We used the data from the 150 participants in the intervention group in the analyses in this study.

### Run4Love mHealth Intervention

The Run4Love intervention consisted of two components: adapted cognitive behavioral stress management (CBSM) courses and physical activity promotions [20]. We adapted the evidence-based CBSM courses on stress management and coping skills to the local context and modified them into 65 items in multimedia formats, including short articles, audio clips, and posters. These items were sent via a self-developed, enhanced WeChat platform for 3 months. In the booster session, 7 materials that were read or listened to the most during the intervention were selected and resent to the participants in the next 3 months after the intervention. The articles were approximately 1300 words and took approximately 5 minutes to read; the audio clips were 5-10 minutes in length; and the posters were pictures with motivational captions, which took <30 seconds to read. Physical activity promotions consisted of goal-setting and information on the guidance and benefits of regular exercise. The enhanced WeChat platform had added functions of automatic information sending, course completion tracking, and weekly personalized feedback. Participants in the intervention group received up to US $2 as financial incentives based on their course content completion via WeChat accounts on a weekly basis.

### Measurement

#### Overview

This study collected data on individuals’ sociodemographic characteristics, patient engagement, and psychosocial outcomes, including depressive symptoms, QOL, and perceived stress. Psychosocial outcomes were assessed at baseline and 3-, 6-, and 9-month follow-ups, collected by research staff using electronic questionnaires on a tablet. Data on patient engagement were collected automatically using the enhanced WeChat platform. Sociodemographic characteristics included age, gender, marital status, sexual orientation, and educational level.

#### Patient Engagement

Patient engagement was assessed through the patient’s completion rate of course assignments and frequency of items completed as these 2 measurements were recommended as reliable measures of patient engagement in mHealth interventions targeting psychosocial outcomes [14] and proven in our previous study [15]. The results from our previous study revealed that these 2 measurements were significantly associated with reduced depressive symptoms at 3 months in the Run4Love intervention. Therefore, we grouped the participants according to these 2 measurements of patient engagement using cluster analysis. A total of 72 intervention items in the form of short articles, audio clips, and posters were delivered to the participants, of which 65 (90%) were sent during the 3-month intervention and 7 (10%) were resent 0 to 3 months after the intervention as a booster. The completion rate was calculated as the percentage of items completed out of 72 by a participant. Items that were clicked by the participants were regarded as completed. The frequency of items completed referred to the total number of times the items were read or listened to by a participant during the 3-month intervention and booster session 0-3 months after the intervention. For example, if a participant read 1 item sent during the 3-month intervention twice and the same item in the booster material twice, the frequency of items completed was counted as 4. As the participants were encouraged to practice the skills for stress management from the CBSM courses repeatedly, the frequency of items completed was used to capture the repetition aspect of patient engagement. Both completion rate and frequency of items completed were automatically tracked by the enhanced mHealth platform. Good reliability of the composite measurement of patient engagement was shown in the study, and the Cronbach *α* was .97.

#### Depressive Symptoms

Depressive symptoms were measured using the CES-D scale, one of the most widely used self-reported questionnaires on depressive symptoms in China [21-23]. The CES-D scale consists of 20 items, such as *I felt depressed* and *I did not feel like eating; my appetite was poor*, and each item is rated on a 4-point Likert scale ranging from 0 (*rarely or none of the time*) to 3 (*most or all of the time*). The CES-D scores range from 0 to 60, with higher scores indicating a higher level of depressive symptoms and 16 being the cut-off point for possible clinical depressive symptoms [24]. Scores ranging from 16 to 20, 21 to 25, and 26 to 60 are considered mild, moderate, and severe depressive symptoms, respectively [25]. Good reliability of the CES-D score was shown in the study, and the Cronbach *α* at baseline and the 3-, 6-, and 9-month follow-ups was .77, .76, .84, and .83, respectively.

#### QOL Measurement

QOL was measured using the World Health Organization Quality of Life HIV short version (WHOQOL-HIV BREF), with 31 items assessing 6 domains: physical, psychological, level of independence, social relationships, environment, and beliefs [26]. Each domain comprises items rated on a 5-point Likert scale. The WHOQOL-HIV BREF scores range from 24 to 120, with higher scores indicating better QOL. The WHOQOL-HIV BREF has been widely used in the Chinese population with HIV and has shown good validity and reliability [27-29]. In this study, the Cronbach *α* for the WHOQOL-HIV BREF at baseline and the 3-, 6-, and 9-month follow-ups was .84, .91, .94, and .94, respectively.

#### Perceived Stress

Perceived stress was assessed using the 10-item Chinese version of the Perceived Stress Scale (PSS-10) [30]. The PSS-10 is the most widely used validated instrument for assessing the perception of stress in Chinese population [31-33]. It assesses the participants’ feelings and thoughts in the previous month (eg, *How often have you been upset because of something that happened unexpectedly?*). The PSS-10 scores range from 0 to 40, with higher scores indicating higher levels of perceived stress. Scores ranging from 0 to 13, 14 to 26, and 27 to 40 are considered low, moderate, and high levels of perceived stress, respectively [34]. In this study, the Cronbach *α* for the PSS-10 at baseline and the 3-, 6-, and 9-month follow-ups was .67, .65, .69, and .65, respectively.

### Statistical Analysis

First, descriptive statistics of demographic characteristics, baseline depressive symptoms, QOL, and perceived stress were presented. Continuous variables with normal distribution were described using mean and SD, and those with skewed distribution were described using median and IQR. Categorical variables were described using numbers and percentages.

Second, hierarchical clustering was used to classify participants into different groups based on the 2 metrics of patient engagement. Cluster analysis is an exploratory classification technique to group participants into different categories based on their similarities in specific metrics. This statistical method helps identify different engagement groups taking different dimensions into account. Hierarchical clustering iteratively merges smaller clusters into larger clusters. The hierarchical clustering procedure was as follows:

1. A similarity distance matrix was constructed by calculating the pairwise distance between different observations. Each observation was assigned to an individual cluster; therefore, each observation represented 1 cluster.
2. The 2 clusters *r* and *s* with a minimum distance from each other were identified.
3. Clusters *r* and *s* were merged, and *r* was replaced with the new cluster. Cluster *s* was deleted, and distances between the new cluster and each of the old clusters were computed.
4. Steps 2 and 3 were repeated until the total number of clusters was 2 [35].

Through clustering, we categorized participants into high- and low-engagement groups based on both measurements of completion rate and frequency of items completed, which could be used to evaluate the effects of different levels of patient engagement on health outcomes. To verify the results of the cluster analysis, we used Wilcoxon rank-sum tests to compare patient engagement between the high- and low-engagement groups along the 2 metrics. In addition, Wilcoxon rank-sum tests were used to examine whether patient outcomes were balanced between the 2 engagement groups at baseline.

Finally, generalized linear mixed effects models (GLMMs) with fixed effects of the engagement groups and time and including time as a random effect were conducted to estimate the trajectories of patient outcomes of depressive symptoms, QOL, and perceived stress, by the 2 engagement groups over time. The random effects of time represented interindividual varying time trends. This allowed for the estimation of variance in the outcomes within and among these time groups. The GLMM allows for the simultaneous analysis of repeated measures in a longitudinal design, thus providing a more accurate estimation of changes in outcomes over time. It also allows for the inclusion of cases with missing data [36,37], making it well-suited for longitudinal data that is likely to have missing values, such as in this study.

In total, 3 GLMMs were conducted to evaluate the relationships between levels of patient engagement and 3 health outcomes over time, adjusting for time and baseline characteristics. The dependent variables were depressive symptoms, QOL, and perceived stress measured repeatedly at baseline and 3, 6, and 9 months, whereas the independent variables were the patient engagement groups (high- and low-engagement group, the latter as reference) and the 4 time points (baseline and 3, 6, and 9 months, with baseline as reference). Baseline characteristics were included in the GLMMs as control variables, including age, gender, marital status, sexual orientation, and education. Only statistically significant characteristics were retained in the final models. The model estimate of the coefficient for engagement groups represented how the trajectories of health outcomes differed between the 2 groups. For example, in the GLMM with CES-D as the dependent variable, a significant negative coefficient suggested that the high-engagement group had a reduction in more depressive symptoms over 9 months than the low-engagement group. Statistical significance was defined as *P*<.05. All analyses were conducted using SPSS (version 25; IBM).

## Results

### Baseline Characteristics

The Run4Love trial recruited 300 participants, and the data from the 150 participants in the intervention group were used in this study (Table 1). The mean age of the 150 participants was 28 years. Most were men (142/150, 94.7%), nonheterosexual (130/150, 86.7%), unmarried (132/150, 88%), and well-educated (98/150, 65.3%) with at least some college education. The mean scores for the CES-D, QOL, and perceived stress were 23.9 (SD 6.4), 77.4 (SD 9.0), and 20.0 (SD 4.4), respectively, at baseline. The average scores of the CES-D scale and perceived stress were at moderate levels. The average QOL score was comparable with that of other people living with HIV [27].

**Table 1:** Baseline characteristics and outcomes of the people living with HIV and elevated depressive symptoms in the intervention group (N=150).

Variables		Values
Age (years), mean (SD)		28.0 (5.8)
**Gender, n (%)**		
	Male	142 (94.7)
	Female	8 (5.3)
**Sexual orientation, n (%)**		
	Heterosexual	20 (13.3)
	Homosexual, bisexual, or uncertain	130 (86.7)
**Education, n (%)**		
	High school or lower	52 (34.7)
	More than high school	98 (65.3)
**Marital status, n (%)**		
	Single, divorced, or widowed	132 (88)
	Married	18 (12)
CES-D^a^, mean (SD)		23.9 (6.4)
QOL^b^, mean (SD)		77.4 (9.0)
PSS-10^c^, mean (SD)		20.0 (4.4)

^a^CES-D: Center for Epidemiologic Studies-Depression.

^b^QOL: quality of life.

^c^PSS-10: Perceived Stress Scale.

### Cluster Analysis

The cluster analysis identified 2 clusters that were distinctively different from each other (Table 2). The first cluster consisted of 72 participants with good compliance to the intervention program, completing an average of 74% (53/72) of intervention items (IQR 0.22) and 82 items (IQR 35.50) when accounting for repeated visits. Specifically, the high-engagement group completed an average of 77% (50/65) of intervention items (IQR 0.23) in the 3-month intervention and 43% (3/7) of items (IQR 0.57) in the booster session. The second cluster consisted of 78 participants with low compliance to the intervention program, completing an average of 15% (11/72) of intervention items (IQR 0.23) in the intervention program and 15 items (IQR 23.25) accounting for repeated visits. Specifically, the low-engagement group completed an average of 17% (11/65) of intervention items (IQR 0.25) in the 3-month intervention and 0 (IQR 1.00) in the booster session. The results of the Wilcoxon rank-sum tests confirmed the significant differences between the 2 cluster groups in both engagement measurements (completion rate and frequency of items completed; *P*<.001), with the high-engagement group having significantly better compliance in both engagement measurements than the low-engagement group, verifying the 2 distinct groups categorized through the cluster analysis. There were no significant group differences in depressive symptoms, QOL, and perceived stress at baseline (CES-D: *P*=.54; QOL: *P*=.45; and PSS-10: *P*=.25), indicating a balance in the outcomes between the 2 engagement groups at baseline.

**Table 2:** Differences in the engagement measurements between the high- and low-engagement groups (N=150).

Engagement metrics		High-engagement group (n=72), median (IQR)	Low-engagement group (n=78), median (IQR)	Wilcoxon rank-sum test	*P* value
**Completion rate**					
	3-month intervention	0.77 (0.23)	0.17 (0.25)	3091.0	<.001
	Booster session	0.43 (0.57)	0.00 (0.14)	3877.5	<.001
	Total	0.74 (0.22)	0.15 (0.23)	3091.5	<.001
**Frequency of items completed**					
	3-month intervention	80 (30.50)	13 (21.25)	3084.0	<.001
	Booster session	3 (4.75)	0 (1.00)	3955.5	<.001
	Total	82 (35.50)	15 (23.25)	3084.5	<.001

### Effects of Patient Engagement on Health Outcomes Over Time

**Figure 1:**
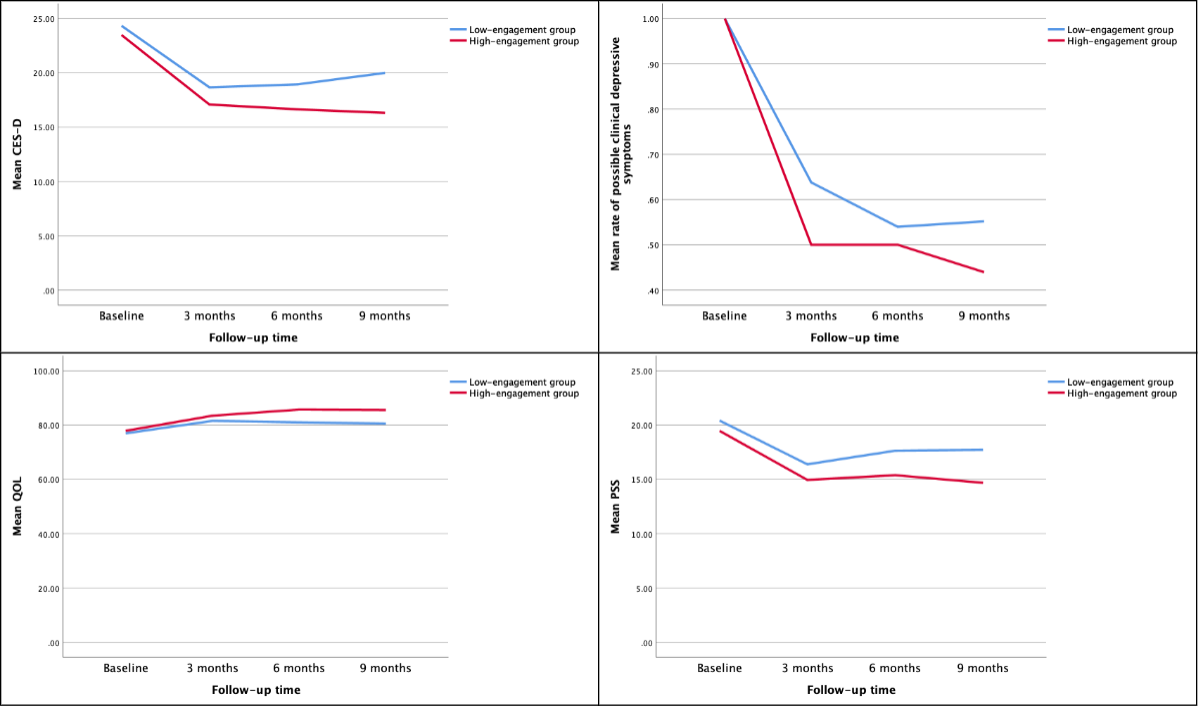
Trajectories of depressive symptoms, quality of life (QOL), and perceived stress over time in high- and low-engagement groups. CES-D: Center for Epidemiologic Studies-Depression; PSS: Perceived Stress Scale.

The trajectories of the 3 outcomes at baseline and 3-, 6-, and 9-month follow-ups are shown in Figure 1, and GLMM results examining the effects of patient engagement on the 3 outcomes are presented in Table 3. Of the 150 participants in the intervention group, 139 (92.7%), 132 (88%), and 133 (88.7%) participants completed the follow-up surveys at 3, 6, and 9 months, respectively. These participants were randomly missing as there were no differences in demographic characteristics and outcomes at baseline between those who completed the follow-up surveys and those who did not. All 3 health outcomes in both groups significantly improved at 3 months immediately after the 3-month intervention (Figure 1). The results of the GLMM (Table 3) showed that the *β* coefficients of the 3 intervention outcomes were all statistically significant, indicating significant between-group differences in these outcomes over time.

**Table 3:** Effects of patient engagement on intervention outcomes at the 3-, 6-, and 9-month follow-ups: results from generalized linear mixed effects models^a^.

Variables				*β* coefficient (SE; 95% CI)		*P* value
**CES-D^b^**						
	Intercept		19.53 (1.86; 15.87 to 23.18)		<.001	
	**Engagement group**					
		High vs low	−1.93 (0.72; −3.34 to −0.51)		.008	
	**Follow-up**					
		3-month follow-up vs baseline	−6.02 (0.94; −7.87 to −4.16)		<.001	
		6-month follow-up vs baseline	−6.11 (0.99; −8.05 to −4.17)		<.001	
		9-month follow-up vs baseline	−5.78 (1.08; −7.91 to −3.65)		<.001	
	Age (years)		.16 (0.06; 0.03 to 0.28)		.01	
	**Education**					
		High school or lower vs more than high school	2.83 (0.75; 1.35 to 4.31)		<.001	
**QOL^c^**						
	Intercept		78.55 (0.90; 76.77 to 80.33)		<.001	
	**Engagement group**					
		High vs low	2.41 (0.93; 0.59 to 4.23)		.01	
	**Follow-up**					
		3-month follow-up vs baseline	5.05 (1.22; 2.65 to 7.44)		<.001	
		6-month follow-up vs baseline	6.01 (1.27; 3.51 to 8.50)		<.001	
		9-month follow-up vs baseline	5.74 (1.34; 3.10 to 8.38)		<.001	
	**Education**					
		High school or lower vs more than high school	−6.62 (0.97; −8.53 to −4.71)		<.001	
**PSS-10^d^**						
	Intercept		20.28 (0.45; 19.39 to 21.17)		<.001	
	**Engagement group**					
		High vs low	−1.72 (0.45; −2.61 to −0.82)		<.001	
	**Follow-up**					
		3-month follow-up vs baseline	−4.25 (0.60; −5.44 to −3.07)		<.001	
		6-month follow-up vs baseline	−3.42 (0.61; −4.63 to −2.22)		<.001	
		9-month follow-up vs baseline	−3.78 (0.65; −5.05 to −2.50)		<.001	
	**Education**					
		High school or lower vs more than high school	1.51 (0.48; 0.57 to 2.44)		.002	

^a^Generalized linear mixed effects models included all participants in the intervention group at all time points. Analyses were adjusted for individual characteristics, including age, gender, marital status, sexual orientation, and education at baseline, and those that did not show significant effects were removed from the final models. Educational level and age remained in the final models.

^b^CES-D: Center for Epidemiologic Studies-Depression.

^c^QOL: quality of life.

^d^PSS-10: Perceived Stress Scale.

The average group difference in CES-D scores between the high- and low-engagement groups was 1.93 (*β*=−1.93; *P*=.008) points over the 9 months, with the high-engagement group having lower levels of depressive symptoms than the low-engagement group. After the initial drastic decreases in both groups during the 3-month intervention (high-engagement group from 23.5 to 17.1 points; low-engagement group from 24.3 to 18.7 points), group differences in depressive symptoms increased in 0 to 6 months after the intervention. As shown in the first graph in Figure 1, there was a slowly rising trend in the CES-D scores at 3, 6, and 9 months (18.7, 18.9, and 20.0, respectively), suggesting some rebound in depressive symptoms in the low-engagement group. In contrast, CES-D scores continued to decrease in the high-engagement group at 3, 6, and 9 months (17.1 to 16.6 and 16.3, respectively). Thus, between-group differences and standard effect sizes (Cohen *d*) in depressive symptoms (CES-D scores) increased over time, with 1.6, 2.3, and 3.7 points and 0.17, 0.23, and 0.34 points at 3, 6, and 9 months, respectively. The high-engagement group experienced a 14%, 4%, and 11% decrease in the rate of possible clinical depressive symptoms compared with the low-engagement group at 3, 6, and 9 months (50% vs 64%, 50% vs 54%, and 44% vs 55%, respectively).

Similar trends were also observed in the other 2 outcome measures. The average group differences in the QOL and perceived stress scores between the high- and low-engagement group were 2.41 (*β*=2.41; *P*=.01) and 1.72 (*β*=−1.72; *P*<.001) points over the 9 months, with the high-engagement group achieving better health outcomes across both measures. After the 3-month intervention, both outcome measures somewhat rebounded in the low-engagement group at 3, 6, and 9 months (QOL: 81.6, 81.0, and 80.5, respectively; PSS-10: 16.4, 17.7, and 17.7, respectively). In contrast, the high-engagement group had either continued improvement or reduced rebound effects at 3, 6, and 9 months in both outcome measures (QOL: 83.5, 85.7, and 85.6, respectively; PSS-10: 15.0, 15.4, and 14.7, respectively). Therefore, similar trends of widening between-group differences in QOL and perceived stress were observed over time at 3, 6, and 9 months (between-group differences in QOL: 1.9, 4.7, and 5.1 points, respectively; between-group differences in PSS-10: 1.4, 2.3, and 3.0 points, respectively). Standard effect sizes (Cohen *d*) in QOL (QOL scores) and perceived stress (PSS-10 scores) both increased over time, with 0.16, 0.37, and 0.39 in QOL and 0.25, 0.39, and 0.51 in PSS-10 at 3, 6, and 9 months, respectively.

Covariates including age, gender, marital status, sexual orientation, and educational level at baseline were adjusted in the 3 GLMMs, and only statistically significant variables were retained in the final models. Educational level was significantly associated with the 3 outcomes, and age was only significantly associated with depressive symptoms. Specifically, compared with those with higher education, participants with lower educational levels (high school or lower) had poorer health outcomes (CES-D: *β*=2.83, *P*<.001; QOL: *β*=−6.62, *P*<.001; PSS-10: *β*=1.51, *P*=.002). Compared with younger participants, older participants were more likely to report higher levels of depressive symptoms (*β*=.16; *P=*.01).

## Discussion

### Principal Findings

This study was among the first efforts to explore the potential time-varying dose–response relationships between patient engagement and various health outcomes over a span of 9 months using 4–time-point measurement data from the Run4Love mHealth intervention. The main finding was that patient engagement had a positive impact on the health outcomes, including depressive symptoms, QOL, and perceived stress, and such impacts were sustained over 9 months after the baseline. In addition, the dose–response relationship was not only sustained in the long term but also increased over time, as there were widening differences in health outcomes between the high- and low-engagement groups.

Both the high- and low-engagement groups benefited from the intervention, but the high-engagement group benefited more from the intervention consistently as the differences in health outcomes between the 2 groups became more pronounced over time. Such sustained and potentially increasing dose–response relationship in the long term has not been reported in previous mHealth studies. The increased effect sizes in depressive symptoms and QOL at 3, 6, and 9 months and increased effect sizes in perceived stress at 3 and 6 months were small to medium. The effect size of perceived stress at 9 months was medium [38]. In the low-engagement group, the intervention effects on health outcomes from 3 to 9 months are consistent with the findings of previous studies reporting rebound effects in mHealth or CBT interventions [39,40]. In contrast, the high-engagement group did not show similar trends; instead, it showed either sustained improvements or fewer rebound effects in all the health outcomes 0 to 6 months after the intervention, resulting in widening differences in these health outcomes between the 2 engagement groups.

Existing literature suggests that intervention effects tend to decrease or diminish over time after the intervention, with some demonstrating rebound effects [39,40]. For example, a face-to-face study found that women with breast cancer in the CBSM intervention group experienced a significant decrease in depressive symptoms immediately after treatment, but the level of depressive symptoms rebounded to baseline at the 1-month follow-up after the intervention [40].

The reasons for the sustained and widening dose–response relationship in this study are many; evidence-based interventions with rigorous design and good implementation are more likely to have sustained dose–response effects. What is missing in the literature on mHealth interventions is whether the momentum continues over time, for example, in 6- or 9-month follow-ups, and whether engagement level plays a role in this momentum. This study adds new evidence to this gap in the literature. Additional research to understand what factors predict patient engagement is also needed.

Our findings also revealed that education and age were important individual characteristics associated with the effects of the intervention, with participants with lower levels of education and of older age having poorer health outcomes. These findings are consistent with the literature [41-43]. To close the digital gap and bridge health disparities, mHealth interventions should be tailored to the needs of these more vulnerable groups, such as older people and those less educated. For example, mHealth interventions should be designed with easy-to-navigate interfaces, bigger fonts, and plain language with engaging multimedia such as pictures, audios, and videos [44].

### Policy Implications

Given the sustained positive impacts of patient engagement on health outcomes found in this study, it is critical to improve patients’ intervention adherence and engagement in both the intervention and subsequent booster sessions in mHealth interventions. There are some effective ways to improve patient engagement as suggested in the literature and evidenced in our Run4Love trial. First, the intervention content needs to be culturally tailored and personalized, which requires formative research and a pilot study. The Run4Love intervention was developed based on extensive formative research and a pilot study [19,45]. We culturally adapted theory-guided and evidence-based CBSM courses, which have been proven effective in relieving depressive symptoms and improving other health outcomes in people who live with HIV [19,45]. Second, the program needs to have superior usability and user experience. Previous studies have shown that perceived usefulness and user-friendly experience are critical for improving mHealth engagement [46,47]. After many rounds of in-depth interviews with patients and the iterative development process, we designed and tailored the intervention platform and formats to meet their needs, such as delivering more appealing multimedia items [45]. With rigorous design and implementation, the participants reported high levels of satisfaction (92%-97%) at all 3 follow-ups in the Run4Love trial [18].

During the intervention, another way to improve patient engagement is to provide timely and personalized feedback. The enhanced WeChat platform automatically sends weekly feedback on the completion status to each participant. In addition to automatic weekly feedback, the Run4Love program also consisted of 5 phone calls made by the research staff at 1 week and 1, 2, 5, and 8 months to address technical challenges and motivate their participation. In addition, the backend platforms of mHealth interventions and wearable devices allow for the collection of passive data on various dimensions of patient engagement, such as physical activities, sleep hours and quality, log-in times, and time and duration of reading, listening to, or watching the intervention items [48-50]. Taking advantage of the easy-to-track user engagement data available in mHealth interventions is critical for process monitoring and quality control of the trials.

### Limitations

There are several limitations to this study. First, the participants in this study were mostly from urban areas, young, and well-educated, and most were nonheterosexual men. Therefore, generalization of the results should be applied with caution. Second, measurement biases may exist in patient engagement. This study only measured patient engagement in CBSM courses but not in physical activity promotions as patient engagement data were not recorded or available in physical activities. However, from the qualitative interviews, we found that most of the engaged participants had a higher engagement in both CBSM courses and physical activities. Therefore, a long-term dose–response relationship between patient engagement in physical activities and intervention outcomes may exist, which needs to be further explored in future research. Moreover, the intervention items were considered as completed when clicked; therefore, we were not able to verify the actual completion or quality of completion. Nevertheless, the patient engagement metrics in this study served as a reliable measurement to assess the dose–response relationship, and the effect of such potential overestimation of patient engagement might only have diluted the observed dose–response relationship [15,51]. Finally, patient engagement in different formats of the intervention content, including short articles, audio clips, and posters, was not differentiated as this was beyond the objectives of this study. Future studies could further explore better measurements of patient engagement in terms of different intervention components and types of multimedia materials in mHealth interventions.

### Conclusions

In conclusion, this study revealed a positive long-term dose–response relationship between patient engagement and 3 psychosocial outcomes in an mHealth intervention using 4–time-point measurement data over 9 months. High- and low-engagement groups showed significant and widening differences in depressive symptoms, QOL, and perceived stress at the 3-, 6-, and 9-month follow-ups in the Run4Love trial. Future mHealth interventions should improve patient engagement to achieve long-term and sustained intervention effects.

## Appendix

Multimedia Appendix 1 CONSORT-eHEALTH checklist (V 1.6.1).

## Abbreviations

CBSM: cognitive behavioral stress management
CBT: cognitive behavioral therapy
CES-D: Center for Epidemiologic Studies-Depression
GLMM: generalized linear mixed effects model
mHealth: mobile health
PSS-10: Perceived Stress Scale
QOL: quality of life
WHOQOL-HIV BREF: World Health Organization Quality of Life HIV short version
